# Genomic analyses provide insights into the genome evolution and environmental adaptation of the tobacco moth *Ephestia elutella*

**DOI:** 10.3389/fphys.2023.1187522

**Published:** 2023-04-19

**Authors:** Jiadan Xu, Bo Li, Zhimin Jiang, Weimin Wang, Yi Yang, Maofa Yang, Xinhai Ye

**Affiliations:** ^1^ China Tobacco Zhejiang Industrial Co., Ltd., Hangzhou, China; ^2^ Institute of Insect Sciences, Zhejiang University, Hangzhou, China; ^3^ College of Tobacco Science, Guizhou University, Guiyang, China; ^4^ Institute of Entomology, Guizhou University, Guiyang, Guizhou, China; ^5^ College of Computer Science and Technology, Zhejiang University, Hangzhou, China; ^6^ Shanghai Institute for Advanced Study, Zhejiang University, Shanghai, China

**Keywords:** comparative genomics, genome evolution, Lepidoptera, *Ephestia elutella*, P450, heat shock protein

## Abstract

*Ephestia elutella* is a major pest responsible for significant damage to stored tobacco over many years. Here, we conduct a comparative genomic analysis on this pest, aiming to explore the genetic bases of environmental adaptation of this species. We find gene families associated with nutrient metabolism, detoxification, antioxidant defense and gustatory receptors are expanded in the *E. elutella* genome. Detailed phylogenetic analysis of P450 genes further reveals obvious duplications in the CYP3 clan in *E. elutella* compared to the closely related species, the Indianmeal moth *Plodia interpunctella*. We also identify 229 rapidly evolving genes and 207 positively selected genes in *E. elutella*, respectively, and highlight two positively selected heat shock protein 40 (*Hsp40*) genes. In addition, we find a number of species-specific genes related to diverse biological processes, such as mitochondria biology and development. These findings advance our understanding of the mechanisms underlying processes of environmental adaptation on *E. elutella* and will enable the development of novel pest management strategies.

## 1 Introduction

Comparative genomics usually provides insights necessary to understand the evolutionary biology of organisms, and it is also a powerful tool to decipher the bridge between genomic characteristics and some specific phenotypes of the species. For insect pests, comparative genomics has significantly advanced our understanding of environmental adaptation mechanisms and the developmental factors potentially associated with pest outbreaks (Wu et al., 2019). For instance, three notable comparative genomics investigations focused on the fall armyworm *Spodoptera frugiperda* provided valuable information on the genetic adaptations related to polyphagia and insecticide resistance, which greatly enhanced our understanding of its rapid global dispersal, invasion, and pest management in China around 2019 (Xiao et al., 2020; Zhang et al., 2020; Gui et al., 2022). Additionally, a comparative genomics study between a generalist and a specialist *Bactrocera* fruit fly elucidated the role of vision and olfaction evolution in feeding habit divergence, potentially inspiring new pest control strategies (Wang et al., 2022). Moreover, genomic analyses of the western flower thrip *Frankliniella occidentalis* answered that why this species has the skills to find, colonize, and survive on various crops (Rotenberg et al., 2020). In a word, comparative genomics provides novel insights into the biological characteristics of insect pests at the sequence level and from the evolutionary perspective, and the findings can be further used to guide the innovative pest management strategies.

The tobacco moth, *Ephestia elutella*, is a destructive pest that consumes a diverse array of stored products, including tobacco, coffee, grain products, and dried fruits (Ashworth, 1993). In the tobacco industry, *E. elutella* has caused significant damage to cured tobacco leaves. Additionally, some insect-derived materials such as dead bodies and excrements, have seriously degraded the quality of tobacco leaves. Although the basic biology and ecology of *E. elutella* are well-documented (Ashworth, 1993) and a high-quality genome assembly is accessible (Yan et al., 2021), no detailed comparative genomics investigation has been conducted to examine the genomic features underpinning this pest’s environmental adaptation and insecticide resistance, which are essential for devising novel pest control strategies. In this study, we employed comparative genomics to probe the genetic bases of the environmental adaptation of *E. elutella*.

## 2 Materials and methods

### 2.1 Orthology inference

Considering genome quality, evolutionary position, and popularity, our comparative genomics analyses contained 20 lepidopteran species, including *Plutella xylostella* [IBG_00646 from InsectBase 2.0 (Mei et al., 2022)], *Cydia pomonella* (IBG_00224 from InsectBase 2.0), *Danaus plexippus* (IBG_00230 from InsectBase 2.0), *Papilio xuthus* (IBG_00614 from InsectBase 2.0), *Thyatira batis* (IBG_00764 from InsectBase 2.0), *Bombyx mori* (IBG_00145 from InsectBase 2.0), *Dendrolimus punctatus* (IBG_00235 from InsectBase 2.0), *Ectropis grisescens* (IBG_00357 from InsectBase 2.0), *Helicoverpa armigera* (IBG_00442 from InsectBase 2.0), *S. frugiperda* (IBG_00715 from InsectBase 2.0), *Chilo suppressalis* (IBG_00117 from InsectBase 2.0), *Cnaphalocrocis medinalis* (IBG_00192 from InsectBase 2.0), *Ostrinia furnacalis* (IBG_00602 from InsectBase 2.0), *Endotricha flammealis* (ilEndFlam1.2 from the Darwin Tree of Life Project), *Hypsopygia costalis* (ilHypCost1.2 from the Darwin Tree of Life Project), *Acrobasis suavella* (ilAcrSuav1.1 from the Darwin Tree of Life Project), *Amyelois transitella* (IBG_00027 from InsectBase 2.0), *Plodia interpunctella* (IBG_00645 from InsectBase 2.0), *Galleria mellonella* (IBG_00397 from InsectBase 2.0), and the tobacco moth *E. elutella* (Yan et al., 2021). OrthoFinder v2.5.3 (Emms and Kelly, 2019) was utilized to identify the orthologous and paralogous genes. For genes with alternative splicing variants, the longest transcripts were selected.

### 2.2 Phylogenetic reconstruction

We used the STAG (Species Tree Inference from All Genes) algorithm (Emms and Kelly, 2018) that integrated in OrthoFinder v2.5.3 to identify genes for phylogenetic reconstruction. Totally, 1,864 orthogroups were used for species tree inference. Multiple sequence alignments (MSA) were inferred for each orthogroup using MAFFT v7.487 (Katoh and Standley, 2013) with L-INS-i model and the species tree was inferred from the concatenated MSAs using IQ-TREE v2.0 (Minh, et al., 2020) with 1,000 replicates for ultrafast bootstrap analysis. The best-fitting model of sequence evolution estimated by ModelFinder that integrated in IQ-TREE v2.0 was used. And we used r8s v1.81 (Sanderson, 2003) to estimate the divergence times between species or clade. Six time points obtained from a previous study (Kawahara et al., 2019) were used to calibrate the tree: Yponomeutoidea: 113–142 million years ago (mya), Tortricoidea: 99–124 mya, Papilionoidea: 87–110 mya, Pyraloidea: 79–104 mya, Noctuoidea: 37–55 mya, and Pyraloidea + Noctuoidea + Geometroidea + Lasiocampoidea + Bombycoidea + Drepanoidea: 90–113 mya.

### 2.3 Gene family expansion and contraction

The CAFE v5 (Mendes et al., 2020) was used to analysis the gene family expansion and contraction, and the results from OrthoFinder and a phylogenetic tree with divergence times were used as inputs for CAFE.

### 2.4 Gene family annotation and analysis

We further annotated some specific gene families related to detoxification, chemoreception, and heat shock protein (Hsp) gene family in the species of our study, because these gene families are crucial for the environmental adaptation of insects. To identify gene models of odorant receptors and gustatory receptors that evolve rapidly (i.e., with high sequence variation), we scanned gene models in genome assembly to avoid missing genes during genome annotation. The proteins sequences of previous reported insect ORs/GRs were first aligned to each genome sequence using Exonerate v2.4.086 (Slater and Birney, 2005). Next, we used a developed pipeline (Karpe et al., 2021) to identify the ORs/GRs in each alignment region. Other genes were annotated using a BITACORA pipeline v1.4.1 (Vizueta et al., 2020) (with Protein model, a BLASTP-based method) and followed by manual confirmation. Lastly, each protein sequence was used for searching against the Pfam-A database by HMMscan v3.3.289 (Prakash et al., 2017) to identify the protein domains. For phylogenetic analysis, the protein sequences were initially aligned using MAFFT v7.487 with L-INS-I model (Katoh and Standley, 2013). After filtering with trimAl v1.2 (-automated1) (Capella-Gutierrez et al., 2009), these sequences were further used to construct a maximum likelihood phylogenetic tree using IQ-TREE v2.0 (Minh et al., 2020) with 1,000 replicates for ultrafast bootstrap analysis.

### 2.5 Identification of rapidly evolving genes and positively selected genes

A total of·2,126 strictly single-copy orthologous genes among ten Pyraloidea insects were used for identification of rapidly evolving genes and positively selected genes. We used Codeml in PAML v4.9 (Yang, 2007) to detect rapidly evolving and positive selection signals on the *E. elutella* branch as we described previously (Yang et al., 2021; Ye et al., 2022). Briefly, we used the branch model in Codeml to detect rapidly evolving genes on the *E. elutella* branch, and a likelihood ratio test (LRT) was performed compare the null model (assuming that all branches have evolved at the same rate) and the alternative model (*E. elutella* branch has a different evolutionary rate). For positive selection identification, we used the branch-site model in Codeml, and also conducted an LRT to compare the null model (sites under neutral or purifying selection) and the alternative model (sites under positive selection on the *E. elutella* branch). Subsequently, *p*-values were computed based on chi-square statistics with a false discovery rate (FDR), and genes with *p*-adjusted value less than 0.05 were identified as rapidly evolving genes and positive selection genes.

### 2.6 Enrichment analysis

GOATOOLS v1.0.6 was used for GO enrichment analyses (Klopfenstein et al., 2018).

## 3 Results

### 3.1 Gene content comparison and genome evolution

**FIGURE 1:**
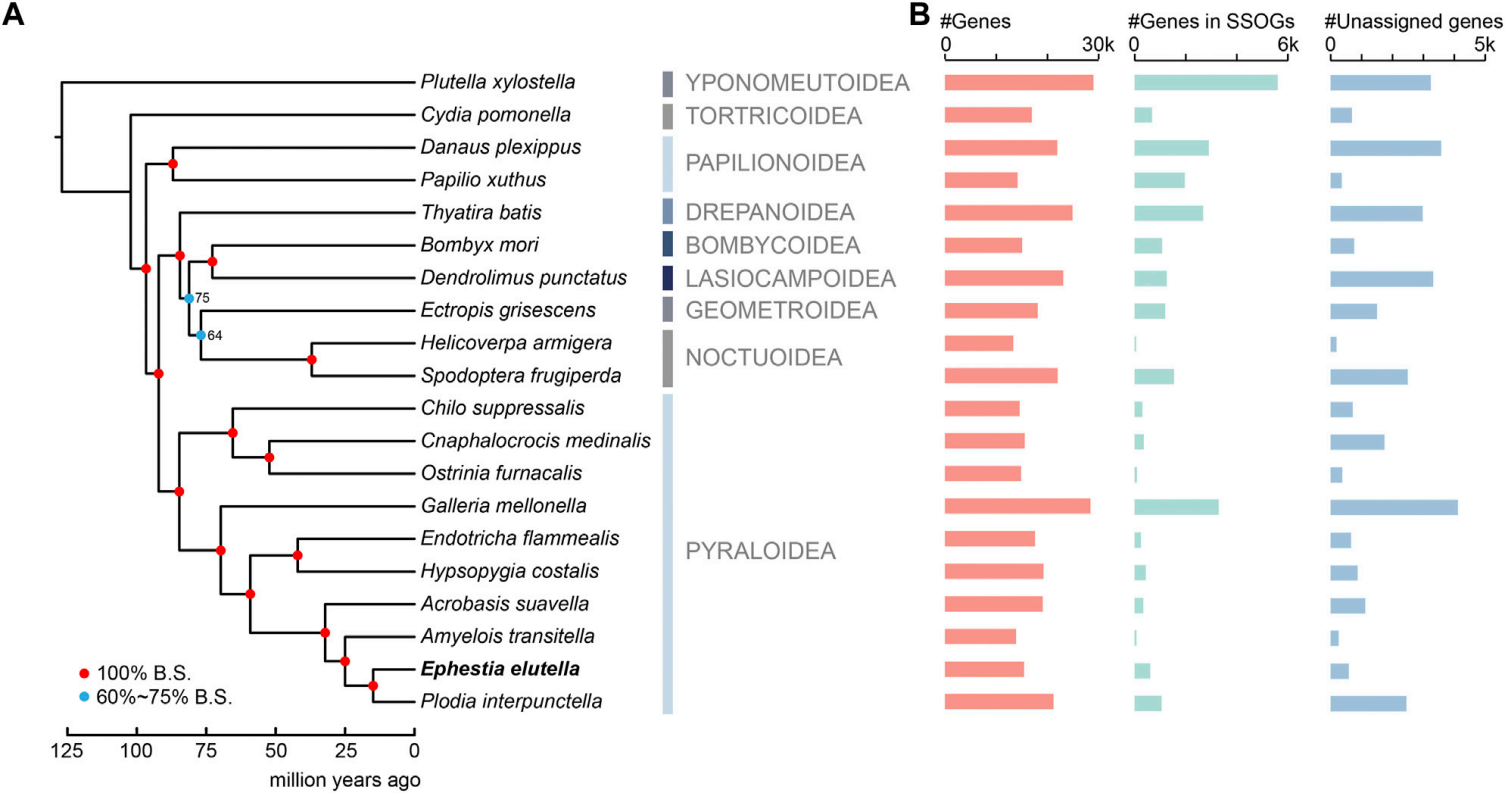
Phylogenetic relationship and orthology comparison of the tobacco moth *Ephestia elutella* and 19 lepidopteran species. **(A)** A maximum-likelihood phylogenetic tree constructed using concatenated protein sequences of 1,864 orthogroups identified by STAG algorithm and 6 calibration points. The color-coding nodes of the phylogenetic tree indicate bootstrap support scores. **(B)** Gene number comparisons of protein-coding genes, genes in species-specific orthogroups (SSOGs) and unassigned genes among the 20 lepidopterans in this study.

We performed comparative genomics analysis among the tobacco moth *E. elutella* and other 19 lepidopteran insects. These species cover several representative lineages of Lepidoptera evolution, with half of them (including *E. elutella*) belonging to the superfamily Pyraloidea, focusing our comparative genomics on this groups (Figure 1A). In addition, our genomic comparison among *E. elutella* and relatively closely-related species could provide key insights into recent adaptive evolutionary features. In total, we identified 22,304 orthogroups (OGs) using OrthoFinder, and of them, 3,896 OGs were present in all analyzed species and 554 strict single-copy OGs were identified. In the tobacco moth *E. elutella*, 10,747 OGs (15,016 genes, 96% of all predicted genes) were clustered. Furthermore, 135 species-specific OGs (SSOGs, 654 genes) and 621 single-copy species-specific and/or orphan genes (i.e., unassigned genes) were found in *E. elutella* relative to other lepidopteran insects (Figure 1B). Based on gene ontology (GO), genes in SSOGs were enrichment for protein dephosphorylation, DNA biosynthetic process (*p* < 0.05, FDR-adjusted; Supplementary Table S1). And the GO terms of unassigned genes were related to mitochondrial processes, imaginal disc growth, and cell-cell junction assembly, but no significantly enriched GO terms were observed for these genes, suggesting diverse functions (Supplementary Table S2). Additional comparative genomics statistics can be found in Supplementary Table S3.

To determine the phylogenetic relationship between *E. elutella* and the other 19 lepidopteran insects, we used a set of genes from 1,864 OGs identified by the STAG algorithm, which was developed to leverage not only strictly single-copy orthologous genes but also the data from multi-copy gene families for phylogenetic analysis (Emms and Kelly, 2018). We found that ten species from the superfamily Pyraloidea clustered together, and the Pyraloidea clade was a sister group to the clade that was composed of Drepanoidea, Bombycoidea, Lasiocampoidea, Geometroidea, and Noctuoidea (Figure 1A). Within the Pyraloidea clade, *E. elutella* was clustered with *P. interpunctella*. The diamond back moth *P. xylostella* (Yponomeutoidea) and the codling moth *C. pomonella* (Tortricoidea) represented the relatively early branches of Lepidoptera. Our divergence time analyses suggested that the Pyraloidea clade diverged from other lepidopteran clusters around 84.7 mya, and the lineage to which *E. elutella* belongs diverged from other Pyraloidea approximately 14.9 mya (Figure 1A).

### 3.2 Global analysis of gene family evolution

**FIGURE 2:**
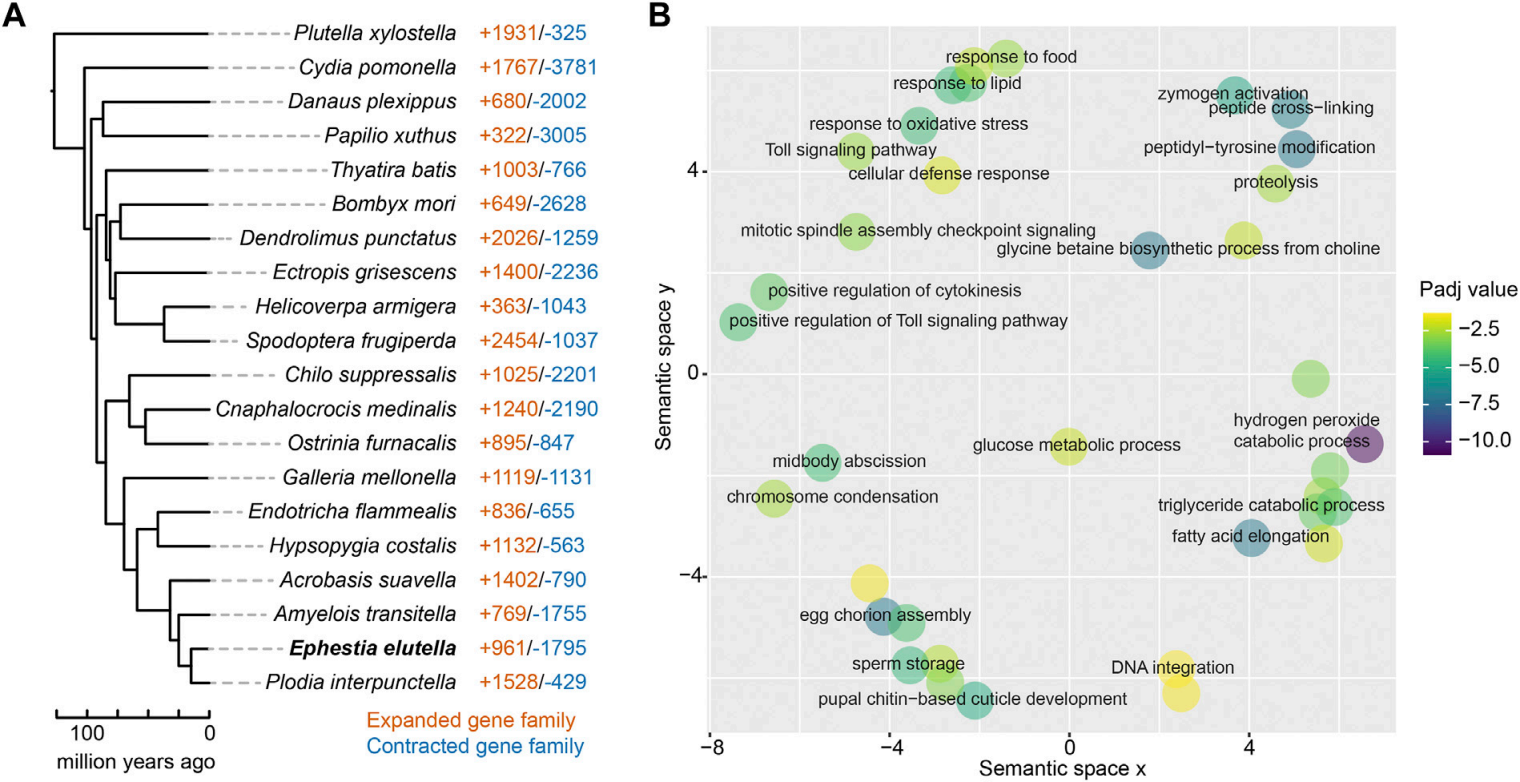
Gene family evolution of *E. elutella*. **(A)** The number of expanded gene families and contracted gene families on terminal branches of the lepidopteran phylogeny that obtained from Figure 1A. **(B)** GO terms of the *E. elutella* expanded gene families are summarized using REVIGO.

We subsequently examined the expansion and contraction of gene families in *E. elutella*, compared with gene families of the common ancestor of *E. elutella* and *P. interpunctella*, and identified 961 expanded and 1,795 contracted gene families, respectively (Figure 2A). GO enrichment analysis revealed that the *E. elutella* expanded gene families are enriched in hydrogen peroxide catabolic processes, eggshell chorion assembly, fatty acid elongation, proteolysis, and the Toll signaling pathway (*p* < 0.05, FDR-adjusted; Figure 2B; Supplementary Table S4). Notably, a total of 75 gene families underwent significant expansion on the *E. elutella* terminal branch (*p* < 0.05). They included some gene families associated with nutrient utilization, such as Serine hydrolase-like protein (OG0000376), Serine protease (OG0000113), Lipase 1 (OG0001370), and Glucose dehydrogenase (OG0000032). In addition, several detoxification-related gene families also exhibited significantly expended signals, including Cytochrome P450 6B1 (OG0000247), Cytochrome P450 4C21 (OG0002002), and ATP-binding cassette sub-family A member 17 (OG0000575). Moreover, we noticed several expended gene families in *E. elutella* that might be related to oxidative stress, including Catalase (OG0000746) and Peroxidase (OG0000106).

Next, we built orthogroup gene trees and summarized the gene duplications over the branches of the species tree, illustrating the overall gene duplication event landscape during Lepidoptera evolution. In total, we identified 2,248 duplication events (related to 7,138 genes) on the terminal branch of *E. elutella*, and GO enrichment analysis showed that these *E. elutella-*branch occurred duplication events were associated with fatty acid biosynthetic process, digestion, pheromone biosynthetic processes, etc., (*p* < 0.05, FDR-adjusted; Supplementary Table S5).

### 3.3 Rapidly evolving genes and positively selected genes

We conducted selective pressure analysis to screen for signatures of rapid evolution and positive selection on the terminal branch of *E. elutella*. To include more single-copy genes and make our analysis more focused on recent evolutionary scale (in Pyraloidea), we utilized 2,126 strictly single-copy orthologous genes among ten Pyraloidea insects for subsequent analyses. Consequently, we identified 229 rapidly evolving genes and 207 positively selected genes in *E. elutella*, respectively (Supplementary Tables S6, S7), with 124 overlapping genes. GO enrichment analyses of these two gene sets did not yield any significantly enriched GO terms (Supplementary Tables S8, S9). And GO annotation suggested that these genes might be involved in various biological processes, including development, cell cycle, stress resistance, and transcription. For example, 11 positively selected genes were associated with regulation of transcription (including transcription factors, such as F-box-like/WD repeat-containing protein and Juxtaposed with another zinc finger protein 1). We also identified three positively selected genes involved in the Hippo signaling pathway, a relatively conserved pathway that controls organ size in animals through regulating cell proliferation and apoptosis (Staley and Irvine, 2012). In addition, three genes related to circadian rhythm displayed positive selection signals, including a Timeless homolog gene. Overall, we suspect that these identified genes with rapidly evolving and positive selection signatures may have facilitated the adaptive evolution of *E. elutella*.

### 3.4 Gene families related to detoxification and chemoreception

Next, we focused on several representative gene families involved in detoxification and chemoreception, given the importance of these biological processes for the environmental adaptation of insects. For insect pests, these processes and related gene families are often research hotspots due to their closed relationship with the studies of insecticide resistance and the development of environmentally-friendly pest control strategies.

**FIGURE 3:**
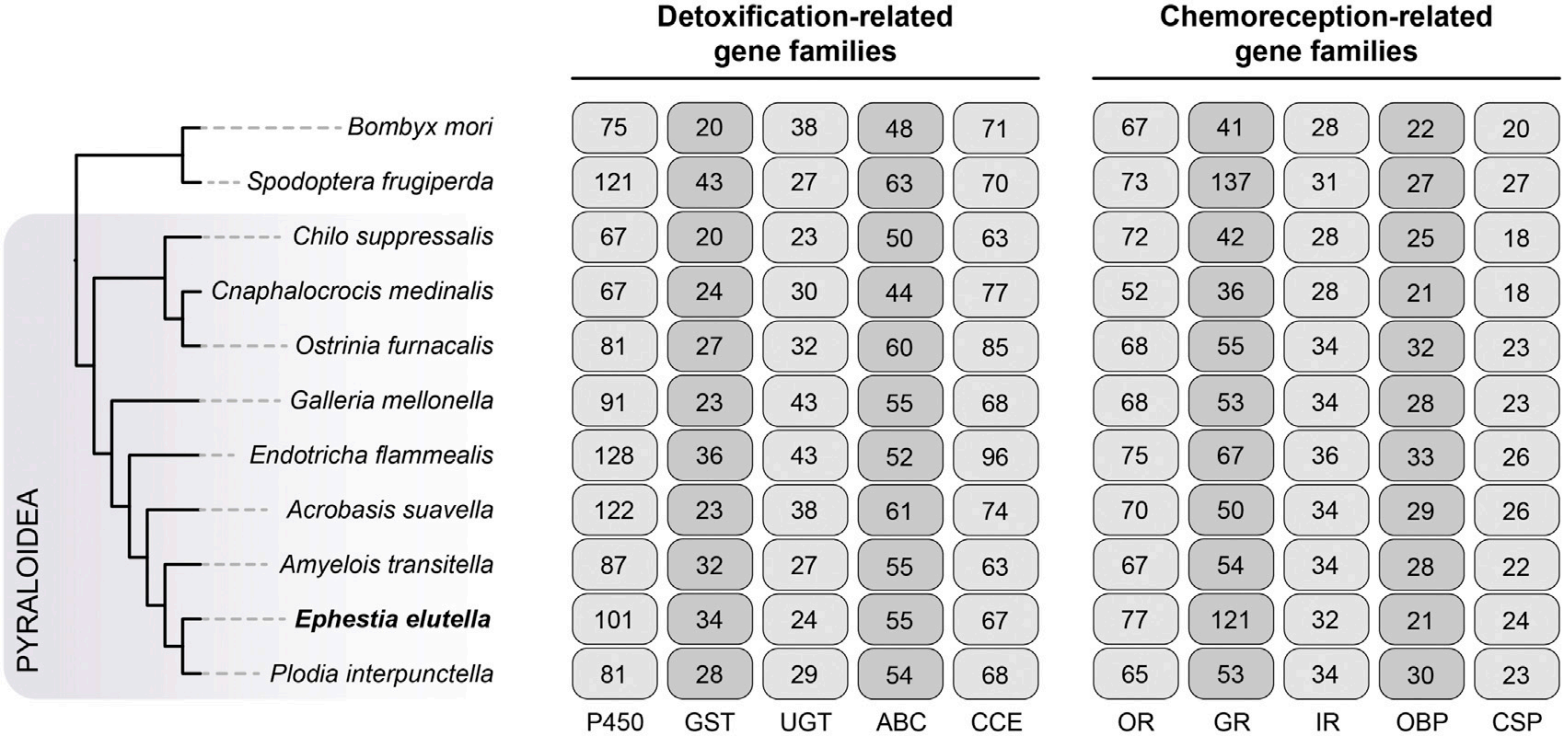
Evolution of chemoreception- and detoxification-related gene families in Pyraloidea. Nine species from Pyraloidea were included in our analysis, and the silkworm *Bombyx mori* and the fall armyworm *Spodoptera frugiperda* were used as the outgroups. The phylogenetic relationship of these species is obtained from Figure 1A. A total of five representative gene families of chemoreception- and detoxification-related gene families were analyzed respectively, and the gene numbers of these gene families are indicated on the right. P450, cytochrome P450; GST, glutathione S-transferase; UGT, UDP glycosyltransferase; ABC, ATP-binding cassette transporter; CCE, carboxyl/choline esterase; OR, olfactory receptor; GR, gustatory receptor; IR, isotonic receptor; OBP, odorant-binding protein; CSP, chemosensory protein.

**FIGURE 4:**
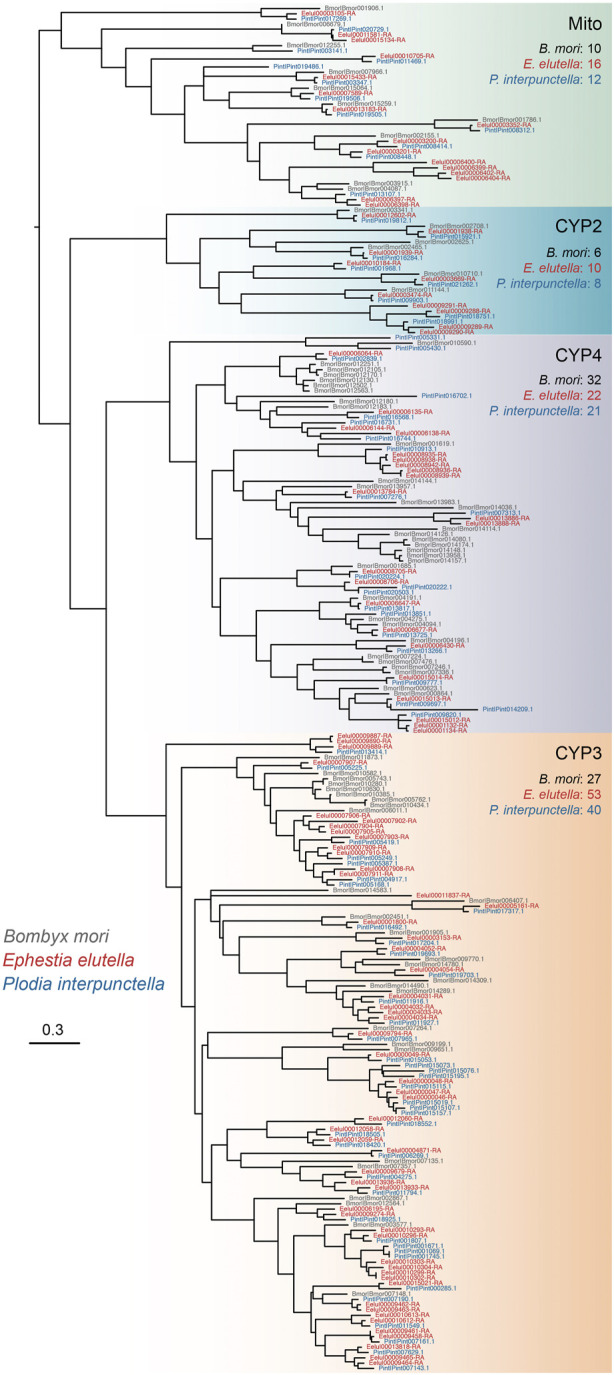
Phylogenetic tree of P450 proteins from *E. elutella*, *P. interpunctella*, and *B. mori*. Four main clades of P450s are indicated, and the gene numbers of these three species in each clade are also shown.

We manually annotated five gene families associated with detoxification in *E. elutella* and ten other lepidopteran species, including cytochrome P450 (P450), glutathione S-transferase (GST), UDP glycosyltransferase (UGT), ATP-binding cassette transporter (ABC), and carboxyl/choline esterase (CCE) (Figure 3). In *E. elutella*, our genome-wide search yielded 101 P450s, 34 GSTs, 24 UGTs, 55 ABCs, and 67 CCEs. When compared with other species in Pyraloidea, *E. elutella* displayed a relatively higher number of P450 genes, surpassed only by *E. flammealis* (128) and *A. suavella* (122). In addition, according to the Pyraloidea phylogeny, the two more closely related species of *E. elutella* (*A. transitella* and *P. interpunctella*) possessed a much smaller number of P450 genes (87 and 80 respectively), suggesting recent P450 gene duplications might have occurred on the *E. elutella* branch (Figure 3). Phylogenetic analysis indicated P450 clan 3 (CYP3) exhibits a large expansion in *E. elutella* compared with those in *P. interpunctella* and the silkworm *B. mori* (Figure 4). We also observed many species-specific gene clusters of *E. elutella* in the P450 tree, inferring the species-specific P450 gene duplications. For chemosensory-related gene families, we mainly focused on olfactory receptor (OR), gustatory receptor (GR), ionotropic receptors (IR), odorant-binding protein (OBP), and chemosensory protein (CSP). Gene annotation and comparative analysis suggested an obvious gene family expansion of GR in *E. elutella* (121 GRs) compared with other Pyraloidea species (from 36 to 67) (Figure 3). Another species with large GR repertoire in our analysis is the fall armyworm *S. frugiperda*, which is a notorious insect pest with an extremely varied range of plant hosts.

### 3.5 Heat shock protein gene family

**FIGURE 5:**
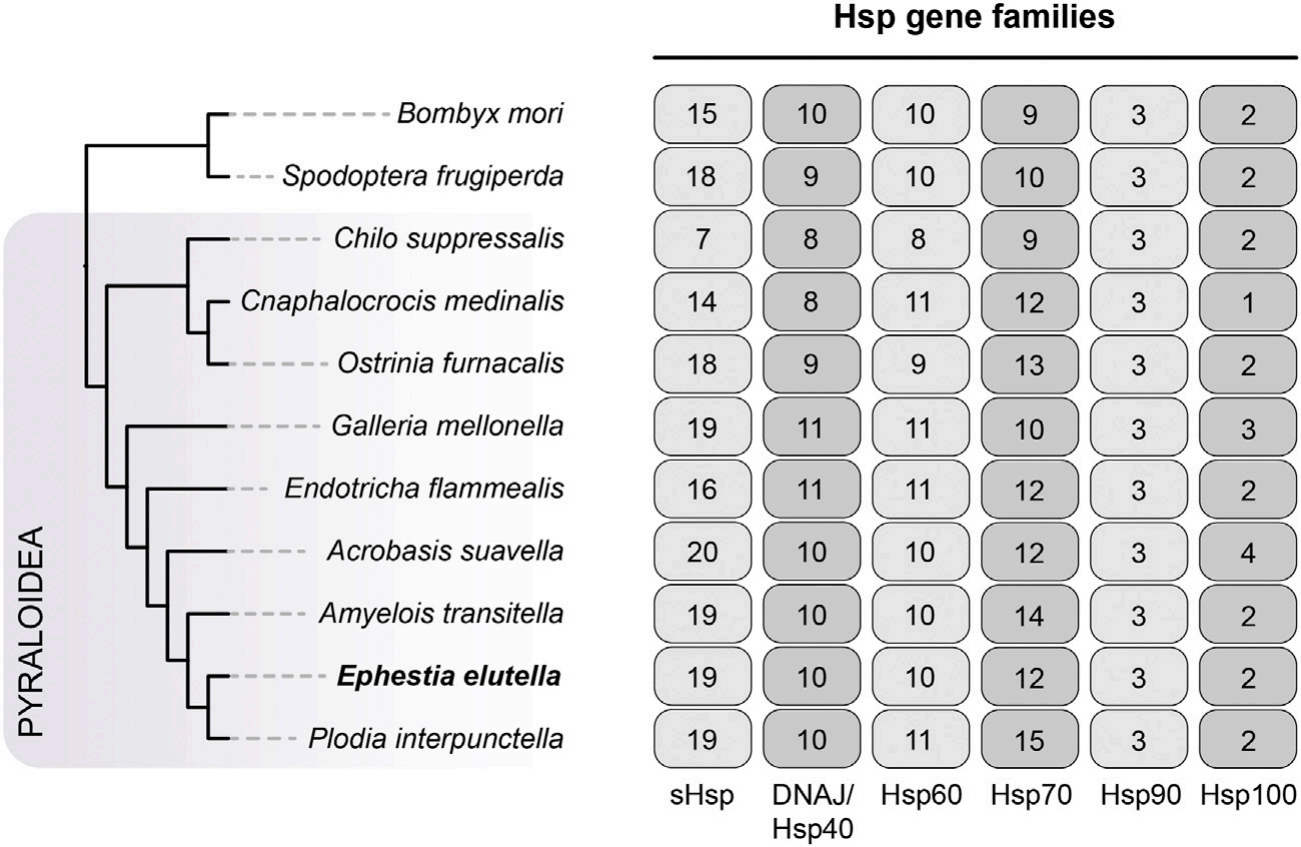
Evolution of Hsp gene families in Pyraloidea. The phylogenetic relationship of the analyzed species (nine Pyraloidea species and two outgroups) is directly obtained from Figure 3. The gene numbers for six Hsp subfamilies, including sHsp, DNAJ/Hsp40, Hsp60, Hsp70, Hsp90, and Hsp100, are indicated on the right.

We also examined the heat shock protein (Hsp) gene family, which includes six Hsp subfamilies, including Hsp40, Hsp60, Hsp70, Hsp90, Hsp100, and small Hsp (sHsp). In *E. elutella*, we identified 19 sHsps, 10 Hsp40s, 10 Hsp60s, 12 Hsp70s, 3 Hsp90s, and 2 Hsp100s (Figure 5). Generally, the copy number of each Hsp subfamily in *E. elutella* was comparable to that of other Pyraloidea insects in our study, suggesting no obvious gene family expansion event occurred in *E. elutella*. And our comparison showed that the *Hsp90* was consistently present in three copies across all 11 lepidopteran insects. Although the copy number is overall conserved among Lepidoptera evolution, we detected two *Hsp40* genes with significant positively selected signals in *E. elutella* (OG0007121 with *p* = 2.05e-14; OG0005866 with *p* = 2.77e-9).

## 4 Discussion

The high-quality genome of the tobacco moth *E. elutella* was released in 2021 (Yan et al., 2021); however, no detailed comprehensive genomic research focused on this pest has been conducted thus far. In this study, we performed a comparative genomic analysis of *E. elutella* and revealed unique aspects of its genomic features. Gene family analyses demonstrated that several digestion-related gene families (e.g., Serine protease and Lipase 1) were expanded in *E. elutella*, which probably facilitates the nutrient metabolism of this pest. In addition, the distinct increase in the copy number of P450 genes (mainly in the CYP3 clan) and species-specific gene duplications in *E. elutella* might enhance the capacity for metabolic detoxification and potentially contribute to the development pest resistance. We also observed that two gene families encoding antioxidant enzymes (Catalase and Peroxidase) were expanded. These genes are typically associated with reactive oxygen species (ROS) scavenging which plays a key role in survival and fecundity in many insect species (DeJong et al., 2007; Diaz-Albiter et al., 2011; Liang et al., 2022). We thus infer that these gene expansion events in *E. elutella* might confer advantages for the biological processes related to reproduction and adaptation to extreme environments. Indeed, diapausing *E. elutella* larvae exhibit strong tolerance to extremes in temperature, humidity and even insecticides (Bell, 1991; Ashworth, 1993). Further functional studies are essential to elucidate the roles of these duplicated P450 genes and antioxidant enzyme genes. Moreover, we noticed the substantially increased number of GR genes in *E. elutella*, which may reflect the broad diet of this moth (including tobacco, cacao beans, cereals, dried fruits, and nuts), similar to other polyphagous insects, such as species from Noctuidae (Cheng et al., 2017; Xiao et al., 2020). The pheromones of *E. elutella* associated with mate attraction and mating behavior have been well-identified (Krasnoff et al., 1984; Krasnoff and Vick, 1984; Ashworth, 1993); however, the specific receptors for these pheromones remain unknown. In this study, we provided sequence resources for chemoreception-related gene repertoires and support future research on the identification of pheromone receptor genes, which might promote the development of novel control approaches for this pest. Additionally, our genome-wide screening highlighted two positively selected *Hsp40* genes in *E. elutella*. Considering the conserved and crucial functions of Hsps in developing tolerance to thermal and various other abiotic stresses among many insects (King and MacRae, 2015; Du, 2018), we speculate that these two *Hsp40* genes may play important roles in the adaptation of *E. elutella* to environmental stresses, such as climate alterations. In conclusion, our genomic analyses enhance the understanding of *E. elutella* biology, and our findings provide a foundation for future research and pest control efforts.

## Data Availability

The original contributions presented in the study are included in the article/Supplementary Material, further inquiries can be directed to the corresponding author.
